# The Nematic Chiral Liquid Crystal Structure of the Cardiac Myoarchitecture: Disclinations and Topological Singularities

**DOI:** 10.3390/jcdd9110371

**Published:** 2022-10-29

**Authors:** Johanne Auriau, Yves Usson, Pierre-Simon Jouk

**Affiliations:** 1 Equipe Biologie Computationnelle et Mathématique, University Grenoble Alpes, CNRS, UMR 5525, VetAgroSup, Grenoble INP, CHU Grenoble Alpes, TIMC, 38000 Grenoble, France; 2 Service de Cardiologie, CHU Grenoble Alpes, CS 10217, CEDEX 9, 38043 Grenoble, France; 3 Equipe Biologie Computationnelle et Mathématique, University Grenoble Alpes, CNRS, UMR 5525, VetAgroSup, Grenoble INP, TIMC, 38000 Grenoble, France; 4 Service de Génétique, Génomique et Procréation, CHU Grenoble-Alpes, CS 10217, CEDEX 9, 38043 Grenoble, France

**Keywords:** anatomy, cardiac myoarchitecture, geometry, topology, liquid crystal, disclination, singularities, remodeling

## Abstract

This is our second article devoted to the cardiac myoarchitecture considered as a nematic chiral liquid crystal (NCLC). While the first article focused on the myoarchitecture of the left ventricle (LV), this new article extends to the whole ventricular mass and introduces the concept of disclinations and topological singularities, which characterize the differences and relationships between the left and right ventricles (RV). At the level of the ventricular apices, we constantly observed a vortex shape at the LV apex, corresponding, in the terminology of liquid crystals, to a “+1 disclination”; we never observed this at the RV apex. At the level of the interventricular septum (IVS), we identified “−1/2 disclinations” at the anterior and posterior parts. During the perinatal period, there was a significant difference in their distribution, with more “−1/2 disclinations” in the posterior part of the IVS. After birth, concomitant to major physiological changes, the number of “−1/2 disclinations” significantly decreased, both in the anterior and posterior parts of the IVS. Finally, the description of the disclinations must be considered in any attempt to segment the whole ventricular mass, in biomechanical studies, and, more generally, for the characterization of myocardial remodeling.

## 1. Introduction

Recent findings show that some biological tissues behave like liquid crystals, opening up a new perspective in understanding the structural mechanisms of living tissues [1,2,3,4]. The enthusiasm of the scientific community towards this approach is explained by the possibility to apply physical conceptual tools of non-living continuous matter (liquid crystals) to biological soft active matter. 

**Figure 1:**
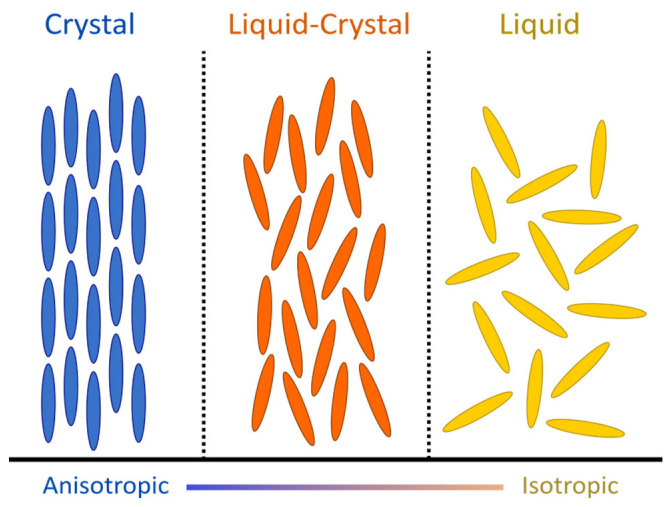
Illustration of the thee states of matter of rod-shaped objects (e.g., myocardial cells and/or myosin molecules). Between the crystal and liquid phase, the liquid-crystal phase is a mesophase sharing some of the properties of the two states of matter, where the molecules will arrange themselves due to their mutual interactions, with a certain degree of order, either positional or orientational while maintaining mobility.

In the human heart, we recently demonstrated with polarized light imaging (PLI) that the myosin myocardial mesh is a biological analogue of nematic chiral liquid crystals (NCLCs), where cardiomyocytes adopt an orientational order and organize themselves along their long axis (Figure 1) [5]. In this previous article, mainly aimed at the cardiology community, we voluntarily focused on the description of the LV alone. Extending this analogy to describing the myoarchitecture of a ventricular mass requires introducing the concepts of topological singularities and disclinations, used in the field of liquid crystals to describe discontinuities in the myocardial mesh. The objective of the current article is to introduce the concept of disclinations, and to find such singularities and disclinations in a ventricular mass [6,7,8,9]. Once these disclinations are found and characterized, the subsequent question is how these behave during hemodynamic changes; explicitly, are they fixed or are they involved in the remodeling process? Our collection of perinatal and early infant hearts allow us to explore one example of such drastic physiological changes. Actually, during the fetal period, the systolic pressure in the right and left ventricles are equal and around 40–45 mmHg. Rapidly after birth, the systolic pressure in the RV falls to 20 mmHg and increases in the LV to 80 mmHg. While anatomical modifications during this transitional period have been finely characterized by other authors [10,11], we here describe the remodeling of the disclinations.

## 2. Material and Methods

### 2.1. Physical Context: Liquid Crystals and Disclinations

**Figure 2:**
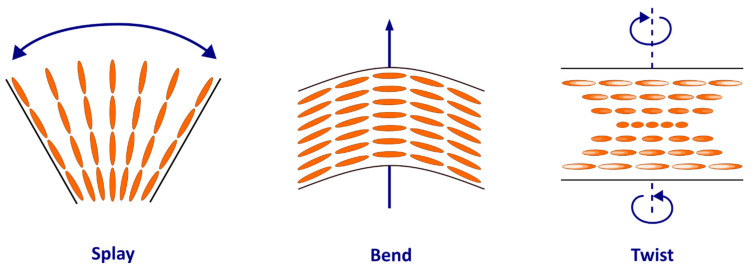
Deformations observed in the nematic phase due to being elastic. Blue arrows represent the type of stress exerted on the molecules causing the deformation.

The term “liquid crystals” was used for the first time in 1900 by F. Reinitzer and O. Lehmann to describe the mesophase between the liquid and solid phases, where molecules have an orientational order (like crystals) but lack a positional order (like liquids) [7,12,13]. In many ways, the myosin mesh shares physical properties with liquid crystals, to the point that it can be considered as an analogue of NCLC. As nematics, myosin molecules are rod-shaped and tend to spontaneously align in parallel along their long axes but not in layers. The average orientation of the molecules (or director) confers onto them an anisotropic structure, unlike liquids, where the molecules have a totally random orientation (Figure 1). Moreover, myosin molecules share with liquid crystals the optical property of birefringence, which makes it possible to extract their 3D orientation and polarized light imaging [14] and confirm it with confocal laser microscopy [15]. Like any structure composed of a mesophase, their spatial configuration depends on the external forces exerted on them, resulting in three possible deformations due to their elastic property (splay, bend, and twist) (Figure 2) [7].

**Figure 3:**
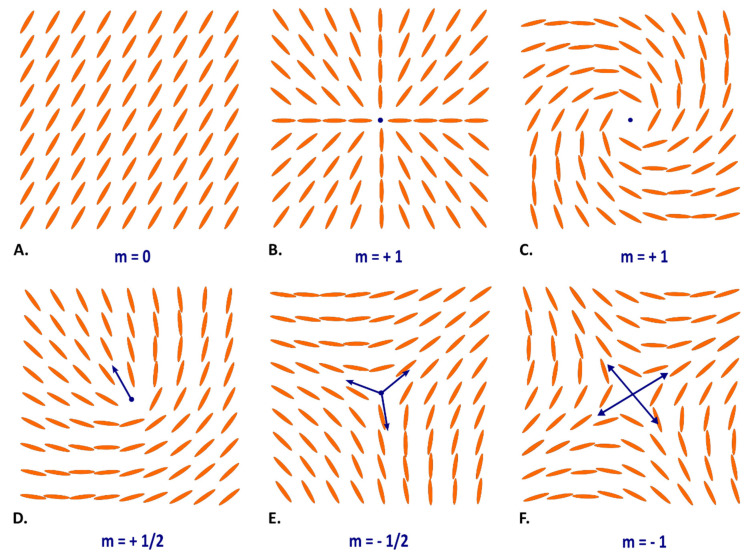
Examples of disclinations (topological singularities) depending on the topological charge, m, measured at the pivotal blue point, (**A**) m = 0, (**B**,**C**) m = +1, (**D**) m = +1/2, (**E**) m = −1/2, (**F**) m = −1. Blue arrows represent the major radial direction pointing out from the singularity (after Tang and Selinger [18]). The method of measurement of the topological charge m is didactically explained in Supplementary Videos S1–S4.

Inside nematic liquid crystals, molecular arrangements are defined by angular correlations between molecules [16]. An abrupt change in the orientation of the director of the molecules generates a topological defect, which is also called disclination when referring to a linear defect [8,17,18]. The high birefringence of liquid crystals and myosin molecules makes it possible to easily identify these defects with PLI. Depending on the strength (or topological charge, m) exerted on the director field of the disclination, different patterns have been described over a wide range of liquid crystals (Figure 3) [7]. This topological charge could either be a positive or negative value, an integer or half-integer. It can be determined by a geometrical process also called the “Volterra Process”, generating a loop (L) that rotates around the singularity in the counterclockwise direction (one full turn = 2π) [6]. By plotting the angle, Θ, traveled by the vectors tangent to the directing field around L, which surrounds the line of disclination, the topological charge, m, which is related to the value of L after one full turn, is defined by the following relation: m = Θ/2 π (Video S1–S4) [8,19,20]. Different configurations are possible for the same value of m.

### 2.2. Myocardial Material

Grenoble-Alpes University hospital owns a legally declared collection of embedded tissue sections collected after autopsies of perinatal and early infant death performed for a diagnostic purpose. Perinatal hearts referred to patients who died before birth of within the first week of life, while early infant hearts referred to patients who died after the first week of life (>7 days). This collection was obtained after a written consent from the parents or guardians at the time of the request for autopsy authorization and for research authorization on normal and abnormal development. The institutional review board of the hospital approved the research protocol and the study was conducted in accordance with the 1964 Declaration of Helsinki and its later amendments. The sample dedicated to research purposes were kept anonymous, but past medical history and patient characteristics were collected. For this study, we selected perinatal and early infant hearts from patients without detectable cardiac anomalies.

### 2.3. Histology, Myocardial Maps, and Disclination Identification

As described in previous publications, heart samples were embedded in methyl methacrylate (MMA) to cancel collagen birefringence, allowing to focus on the birefringence of myosin molecules [14,15,20]. This process was very long and took on average between 8 and 10 weeks per heart, depending on its size. For every heart, a series of thick sections (500 µm) were cut with a rotary microtome along the short axis of the heart, with a loss of 500 µm of material due to the thickness of the wire, resulting in a spacing of 1 mm between sections. Each section was secondarily polished before extracting the 3D-orientation information of the myosin molecules with PLI techniques. The time required for each section for these three steps (section, polishing, and acquisition) was approximately two hours. We identified the mean orientation (director) of all myosin molecules contained in a voxel of 90 × 90 × 500 µm^3^ (around 500 myocardial cells) and expressed this information by two angles in a Cartesian system: the azimuth angle and the elevation angle. Representation of this orientation information was obtained using streamline maps with an LIC texture (Videos S5 and S6) or false color maps for the values of the azimuth angles or elevation angles [21].

Disclinations were identified by a visual analysis of each heart section, using different representation maps to maximize the visual characteristic of each disclination.

### 2.4. Patient’s Characteristics

Eighteen patients were included in our series. Among them, 11 (61%) died during pregnancy (after 22 weeks of gestation) or during the first week of life, and 7 (39%) died after the first week of life. Age at death was missing for two patients, but we were able to classify them reliably as perinatal hearts, given their small heart size. The number of heart sections obtained with the rotary microtome varied from 26 to 51. Section “0” referred to the most basal section, while the highest number referred to the most apical section. Approximately, the higher the number of heart sections, the larger the heart size. Table 1 summarizes the available data according to whether patients died before or after 1 week of life.

## 3. Results

### 3.1. Disclination Identification

**Figure 4:**
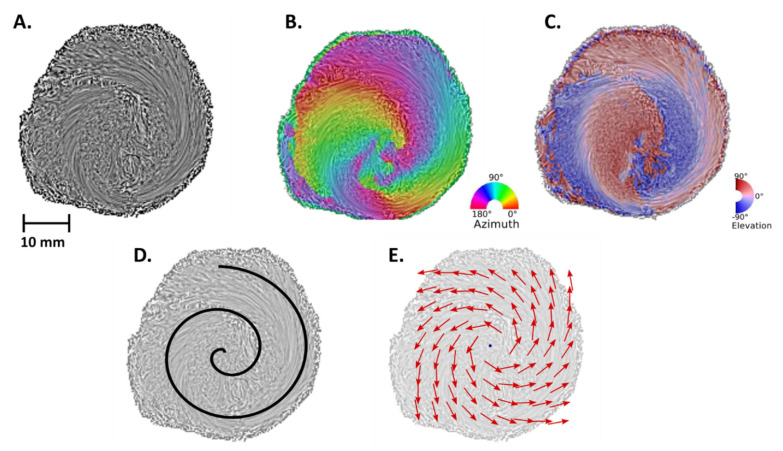
Left ventricular apex and +1 disclination. Apical sections of a perinatal heart (29 gestational weeks). The LV vortex (“+1 disclination”) is observed in the LIC map (**A**), in the azimuth map (**B**), and in the elevation map (**C**). The spiral (**D**) and the vector field (**E**) are superimposed at A, in order to help the reader to visualize the “+1 disclination”.

**Figure 5:**
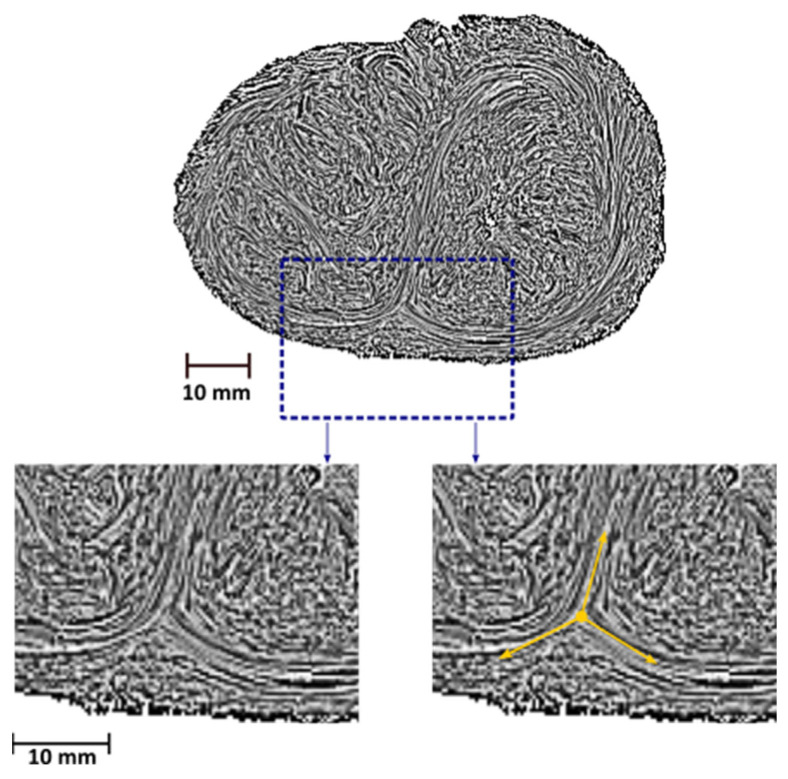
**Example of a “−1/2 disclination” located in the posterior part of the IVS.** Short-axis section of a perinatal heart (4 days of life) at the junction of the apical third and the median third in the streamline map with an LIC texture. Yellow arrows are superimposed to help the reader visualize the “−1/2 disclination”.

We identified on the compact myocardium only two types of disclinations. The first was a disclination of order m = +1 (noted “+ 1 disclination”), identified on the LV apex and illustrated in Figure 4. The second was a disclination of order m = −1/2 (noted “−½ disclination”), identified in the anterior and posterior parts of the IVS, at the connection of the right and the left ventricles (Figure 5).

### 3.2. Left and Right Ventricular Connections to the IVS and “−1/2 Disclination”

**Figure 6:**
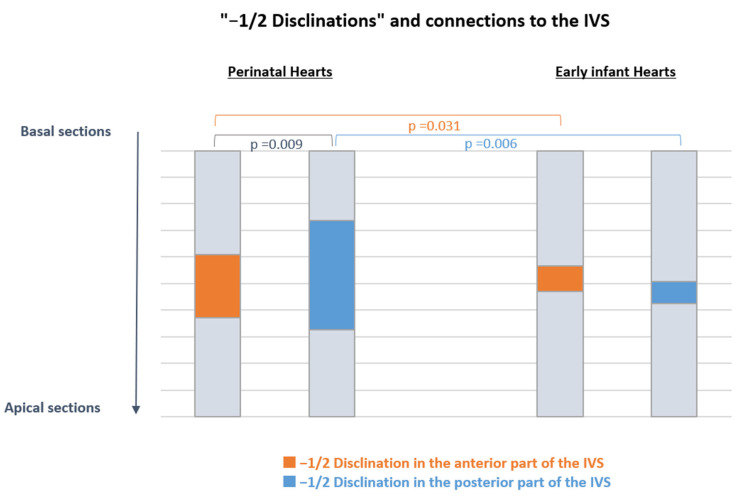
Normalized distribution of the “−1/2 disclinations” in perinatal and early infant hearts. In perinatal hearts, there are more “−1/2 disclinations” in the posterior part of the IVS than in the anterior part of the IVS. In early infant hearts, there are less “−1/2 disclinations” than in the perinatal hearts, whether in the anterior and posterior parts of the IVS. There was no variation in the mean position of the “−1/2 disclinations” along the apico-basal axis between perinatal and early infant hearts.

As described above, the anterior and posterior connections between the left and right ventricles with the IVS were analyzed in each heart section of the patients using streamline maps with an LIC texture. We identified connections with the characteristic of a “−1/2 disclination” in both groups and reported them according to their apico-basal level (Figure 6). After normalizing the positions with respect to the number of sections, we observed more “−1/2 disclinations” in perinatal hearts compared to early infant hearts, whether on the anterior or posterior parts of the IVS (23.7% vs. 10.1%, *p* = 0.031, and 41.7% vs. 9.4%, *p* = 0.006, respectively). This is also true in absolute values. While the total number of “−1/2 disclinations” observed among the 11 hearts of the perinatal group was 238 (mean value of 21.6 disclinations per heart), the total number of “−1/2 disclinations” observed among the 7 hearts of the early infant group was 51 (mean value of 7.3 disclinations per heart).

In perinatal hearts, the number of “−1/2 disclinations” was higher in the posterior than anterior parts of the IVS (23.8% vs. 41.7%, *p* = 0.009), while there was no difference in early infant hearts (10.1% vs. 9.4%, *p* = 0.8). There was no variation in the mean position of “−1/2 disclinations” along the apico-basal axis between perinatal and early infant hearts.

### 3.3. Apex and “+1 Disclination”

**Figure 7:**
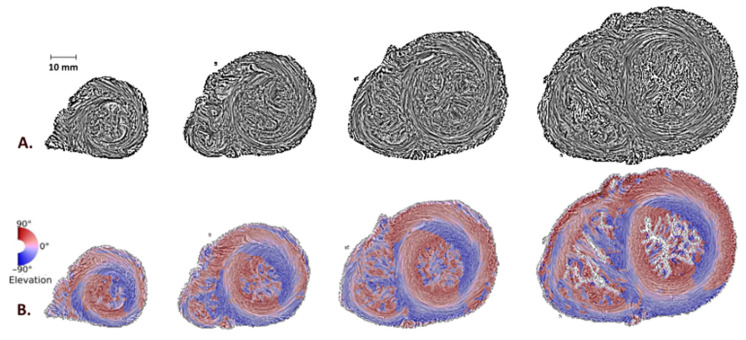
Short-axis sections of a perinatal heart (7 days of life) from the apical to the middle level just under the papillary muscles, using streamline maps with an LIC texture (**A**) or elevation maps (**B**). While the LV apex takes on a vortex shape, this is not the case for the RV apex.

While for all patients the LV apex distinctly presented a vortex shape, corresponding to a “+1 disclination”, it was evident that the shape of the RV apex was different and not that expected of a vortex. This observation was constant regardless of the perinatal or early postnatal period. Figure 7 illustrates with streamline and elevation maps the shape of the left and right ventricular apices, highlighting the absence of the “+1 disclination” at the RV apex.

## 4. Discussion

After demonstrating that the architecture of the LV behave like a liquid crystal, this second article of our series adds the concept of disclinations to the analysis of the whole compact ventricular mass, especially in the IVS [5]. This work is fully in line with the current dynamics of research on soft matter in physics and biophysics [22,23]. These two articles are the first to interpret heart anatomy within the frame of liquid crystal structures.

The pioneering work of the biologist Yves Bouligand in the 1970s described stabilized liquid crystals in biology [24,25]. Since then, it has been demonstrated that the nematic structures observed in biology could also be dynamic [1,2,3,4]. In this article, we extend this dynamic characteristic to the myocardium. The remodeling of the nematic chiral cardiac myoarchitecture due to drastic perinatal hemodynamic changes is exerted through variations in pressure and volume, as is the case for any other physical constraints reported in previous examples in the field of liquid crystals [13,16]. Anatomists and cardiologists have well described the progressive curvature of the IVS, from flat at birth to curved with RV-pointing convexity after postnatal adaptation [11]. We add to this macroscopic statement a myoarchitecture description: the progressive decrease in “−½ disclination” in the IVS.

In contrast, during the postnatal adaptation, we did not observe variations of the myoarchitecture of the two apices: there is a constant topological singularity at the LV apex whereas there is constantly none at the RV apex.

Regarding the LV apex, the vortex appearance is a constant feature of all patients, regardless of size and age, and corresponds to a “+1 disclination”, as illustrated in Figure 3C and Figure 4. We have demonstrated in our previous article that this spiraling aspect is a consequence of the alignment of interconnected chains of myocardial cells or “fibers” as geodesics on a nested set of toroidal surfaces [5].

Regarding the RV apex, we have never observed the appearance of a vortex and other disclinations. While these major differences between the right and left apices are widely acknowledged, to this day, it still lacks a scientifically demonstrated model of the whole myoarchitecture of the RV taking into account these morphological differences [10,11,26,27,28,29,30]. Indeed, the RV does not present any symmetry of revolution, which was an essential characteristic for the validation of the geodesic properties of the LV “fibers”. Actually, there are many conjectures about the myoarchitecture of the RV, but their demonstrations remain impossible in the absence of suitable mathematical tools and sufficient series with a high spatially quantitative resolution. However, the present study takes our conjecture formulated in 2000 one step further [14]. Let us remember this conjecture, which was an extension to the RV of Streeter’s model of the LV, namely, that “fibers are running like geodesics on a nested set of warped pretzels (or double torus). Such a pretzel looks like two torus joined side-by-side” [14]. In such a conjecture, we extend to the RV the nematic chiral myoarchitecture already demonstrated for the LV but not yet for the RV. The study of disclinations in the IVS that we performed in this article reinforces this conjecture, providing an additional argument in favor of the chiral nematic structure of the right ventricular myocardium. The physics of liquid crystals teaches us that at the level of the entanglement of two nematic chiral liquid crystals, “−½ disclinations” are generated [7]; that is what we identified in the IVS, namely, the junction area between the left and right ventricles. The biomechanical consequences of the modification of the disclination pattern during postnatal adaptation are difficult to explore without a solid and validated model of the myoarchitecture of the whole RV. This is what we are working on, using liquid crystal physics and symplectic geometry as tools.

## 5. Conclusions

The purpose of this article was to introduce the concept of disclinations in the description of the myoarchitecture of a compact ventricular mass, to promote these physics concepts in the fields of cardiology and anatomy. We gathered all the conditions that allowed us to identify the disclinations in cardiac myoarchitecture: an adequate sample collection and adapted digitized microscopic methods. While this inaugural work is somewhat marginal because of the technical constraints of PLI and being limited to using perinatal and early infant hearts, it must not be limited to that; it concerns the bulk of cardiology. Indeed, promising resolution improvements in routine cardiac imaging modalities, such as cardiac MRI or CT scans, will surely be allowed within a few years, thereby helping to apply these physical concepts to normal and pathological adult hearts. We are reasonably certain that, in the future, comprehensive methods for understanding and managing pathological cardiac conditions will have to integrate studies on the dynamics of disclinations in a ventricular mass. Describing the disclinations in a ventricular mass will become the standard for all future work on the segmentation of different domains of the ventricular mass and subsequent biomechanical analyses.

## Figures and Tables

**Table 1:** Anthropometric data.

	Perinatal Hearts (>22 Weeks of Gestation or <1 Week of Life)	Early Infant Hearts (>1 Week of Life)
**Total, n**	11	7
**Sex, n** **Female** **Male** **Missing data**	4 5 2	2 4 1
**Gestational age, weeks ** **Median *(min–max)***	38 *(29–41)*	39 *(33–40)*
**In utero death, n**	5	*NA*
**Weeks of life when born alive ** **Median *(min–max)***	0.3 *(0.1–0.4)*	30 *(4–80)*
**Ventricular weight, g** **median *(min–max)***	14.1 *(7.6–19.2)*	34 *(11.7–46.5)*
**Number of cardiac sections, n** **Median *(min–max)***	32 *(26–40)*	42 *(31–51)*

## Supplementary Materials

The following supporting information can be downloaded at: https://www.mdpi.com/article/10.3390/jcdd9110371/s1, Supplementary data videos, Video S1–S4: Principles of the measurement of the topological charge m of a disclination. Video S1: Example of a “+1 disclination”. When the black rotor completes a full turn (2π) around the pivotal point where is calculated the disclination, the green double headed segment also turns of 360° (2π) in the same direction m = 2π/2π = 1. Video S2: Example of a “−1/2 disclination”. When the black rotor completes a full turn (2π) around the pivotal point where is calculated the disclination, the green double headed segment turns only of 180° (−π) in the opposite direction m = −π/2π = −½. Video S3: Example of a “−1 disclination”. When the black rotor completes a full turn (2π) around the pivotal point where is calculated the disclination, the green double headed segment turns of 360° (−2π) in the opposite direction m = −2π/2π = −1. Video S4: Example of a “+1/2 disclination”. When the black rotor completes a full turn 360° (2π) around the pivotal point where is calculated the disclination, the green double headed segment turns only of 180° (π) in the same direction m = π/2π = ½. Video S5: Stack of short-axis sections from apex to the base of a perinatal heart in streamline map with LIC texture (33 gestation weeks, died 6 days after birth). This stack makes it possible to observe the disclinations in the sections. There is no actual dynamic motion, this feeling is created by the progressive change of orientation from one section to the other. Video S6: Stack of short-axis sections from apex to base of an early infant heart in streamline map with LIC texture (40 gestation weeks, died 9 months after birth). There is no actual dynamic motion, this feeling is created by the progressive change of orientation from one section to the other.
